# Genetic assessment of residual feed intake as a feed efficiency trait in the Pacific white shrimp *Litopenaeus vannamei*

**DOI:** 10.1186/s12711-017-0334-1

**Published:** 2017-08-04

**Authors:** Ping Dai, Sheng Luan, Xia Lu, Kun Luo, Xianhong Meng, Baoxiang Cao, Jie Kong

**Affiliations:** 1 Key Laboratory for Sustainable Utilization of Marine Fisheries Resources, Ministry of Agriculture, Yellow Sea Fisheries Research Institute, Chinese Academy of Fishery Sciences, Qingdao, 266071 China; 2 Function Laboratory for Marine Fisheries Science and Food Production Processes, National Laboratory for Marine Science and Technology, Qingdao, 266235 China

## Abstract

**Background:**

Residual feed intake (RFI) was investigated as a measure of feed efficiency in a breeding population of *Litopenaeus vannamei*. Shrimp from 34 families were housed individually and feed efficiency and growth traits were recorded during two successive growth periods. The objectives of this study were (1) to estimate the heritability of RFI and related traits, including feed efficiency ratio (FER), average daily gain (ADG) and daily feed intake (DFI), (2) to determine the relationships between RFI and other traits, and (3) to evaluate the variation of these traits across two growth periods.

**Results:**

Shrimp displayed large inter-individual variation in RFI, FER, ADG and DFI during each growth period. Heritability estimates of all these traits during both periods reached high values (0.577 ± 0.232 to 0.707 ± 0.252). RFI showed weak and no genetic correlations with ADG during the two growth periods between days 1 to 21 (0.135 ± 0.204) and 22 to 42 (–0.018 ± 0.128), respectively, but high positive genetic correlations with DFI (>0.8). Weak and moderate negative genetic correlations were observed between RFI and FER during the two periods (–0.126 ± 0.208 and –0.387 ± 0.183). As evidenced by the high genetic correlations between the two periods for each trait (>0.6), trait performance of the shrimp tended to be consistent across periods.

**Conclusions:**

For the first time, accurate measurement of individual feed efficiency on a large scale was achieved in shrimp. Although the estimated heritability reported here for RFI may be overestimated, it is a heritable trait in *L. vannamei* that can be improved by genetic improvement. For *L. vannamei*, the biggest potential advantage in using RFI as a measure of feed efficiency is that it is independent of growth rate, and thus genetic selection on RFI has the potential to improve feed efficiency and reduce feed intake, without compromising growth performance.

**Electronic supplementary material:**

The online version of this article (doi:10.1186/s12711-017-0334-1) contains supplementary material, which is available to authorized users.

## Background

The Pacific white shrimp, *Litopenaeus vannamei*, which is native to Central and South America on the west coast of the Pacific Ocean, has become the primary cultivated shrimp species in the world [1]. To date, it accounts for more than 80% of total shrimp production in China. In intensive shrimp culture, feed cost usually accounts for 50 to 60% of the total production costs [2] and is a major constraint on profitability for shrimp production enterprises. Improvement in feed efficiency would contribute to the economic sustainability of farmed shrimp production and to a reduction in the environmental load, and, therefore, this trait should be included in the breeding objective of shrimp selection programs. In poultry and livestock, feed efficiency has been significantly improved by both genetic and non-genetic approaches [3].

The most commonly used measures of feed efficiency are feed efficiency ratio (FER) and residual feed intake (RFI) [4]. FER is defined as the ratio between body mass gain and amount of feed consumed. In general, the distribution and irregular statistical behaviour of such ratio measures make their selection difficult [5]. For example, it is not possible to determine whether an improvement in FER results from a decrease in feed intake, an increase in weight gain, or both. RFI was first used in beef cattle by Koch et al. [6] and later introduced in poultry [7] and fish [8, 9]. RFI is defined as the proportion of feed intake that is not accounted for by expected requirements for body weight maintenance and production. A low or negative RFI value stands for high feed efficiency, while a high or positive RFI value indicates low efficiency. As a linear trait, RFI has been reported to be independent of growth, both phenotypically and genetically [10–12]. Thus, selection for reduced RFI may not have detrimental effects on animal growth or size, which is the main advantage of using RFI as a measure of feed efficiency trait instead of FER.

In poultry and livestock industries, use of RFI as a measure of feed efficiency in animal production has raised much interest. To investigate the effectiveness of selecting for feed efficiency, much of the work has concentrated on quantifying variability in RFI and determining how much of this variability is genetic [13]. Most heritability estimates published for terrestrial agricultural animals show that RFI is moderately to highly heritable [14–18] and direct selection aimed at improving this trait for multiple generations has proven effective [19, 20]. Regarding aquatic species, almost all studies on RFI have been carried out in fish and have revealed pronounced genetic variability [8, 9, 21]. To date, few studies have analyzed RFI in shrimp. Moreover, understanding the relationships between RFI and other performance traits is important to evaluate the potential impact of genetic selection for this trait. To accelerate the use of RFI in breeding programs of *L. vannamei*, it is necessary to carry out the genetic evaluation of this trait.

As a prerequisite for evaluating feed efficiency, accurate measurement of individual feed intake during a given period is required but producing such individual data on a large scale in shrimp is a major challenge, since shrimp are usually farmed in large groups. Individual feed intake of fish reared in groups is measured by X-ray technology but the repeatability between measurements is low (from 0.09 to 0.32) and thus repeated measurements are necessary to buffer day-to-day variations [9, 22]. Moreover, Quinton et al. [23] showed that the heritability of feed efficiency estimated by this method is low (6 ± 10%). In addition, this method is labor-intensive and time-consuming when large-scale and frequent measurements are required. Another solution for measuring individual feed intake is to house the animals individually, which has been successful in fish [21, 24, 25]. Such individual measurement of feed intake over a period allows individuals that use their feed more or less efficiently to be identified. A feeding test is often conducted over weeks to months for different species. Because of the potential mortality of shrimp, a testing period of several weeks is reasonable [8, 9, 21, 26]. Furthermore, given the importance of the grow-out period during *L. vannamei* production, a test during this period is preferable.

In this study, we estimated feed intake at the individual level by housing shrimp individually during the grow-out period and investigated RFI as a measure of feed efficiency in the breeding population of *L. vannamei*. The specific objectives of this study were (1) to estimate the heritability of RFI and related traits, including FER, average daily gain (ADG), and daily feed intake (DFI), (2) to determine the relationships between RFI and other traits, and (3) to evaluate the variation of these traits across two growth periods.

## Methods

### Ethical statement

This research was approved by the Animal Care and Use committee at the Yellow Sea Fisheries Research Institute, Chinese Academy of Fishery Sciences. We used the Pacific white shrimp (*L. vannamei*), which is native to Latin American countries and was introduced into China for farming in the late 1990s. It is neither an endangered nor a protected species in any of these countries. All experiments carried out in this study complied with the Law of the People’s Republic of China on the Protection of Wildlife (http://www.china.org.cn/english/environment/34349.htm).

### Shrimp production and trial management

In 2012, a base population was derived from several commercial *L. vannamei* strains (founder population) using an incomplete diallel cross experiment. Subsequently, we produced closed and discrete generations yearly according to a nested mating design and a standardized family production procedure. In 2015, more than 100 full-sib families were produced in generation 3 (G3) from 132 sires and 134 dams. All breeding programs were performed at the Mariculture Research Station of the Yellow Sea Fisheries Research Institute, Chinese Academy of Fishery Sciences (Qingdao, China).

There was an age difference of about 20 days among the families in G3 due to different mating and spawning times, which resulted in growth performance varying among families. Thirty-four families produced by 31 sires and 31 dams were chosen for the feeding test based on a high level of survival at three months. When the smallest shrimp reached 4 cm, 18 individuals were randomly collected from each family and evenly divided into three groups (n = 6 individuals per group). The experiment was conducted in the aquatic housing systems (Haisheng Biotech, Shanghai, China) that were constituted of many independent culture tanks (200 mm × 100 mm × 150 mm) with a water volume of 1.8 L. The bottom of each tank was equipped with a special mesh that allowed the faeces to drain but not the unconsumed feed. These systems work with re-circulating water and have temperature-control sets, UV lights and aeration pumps.

Each shrimp was reared in one tank and three groups of each family were allocated to three housing systems in a randomized complete block design. First, the shrimp underwent a five-day adaptation to the experimental environment. During the feeding test, the shrimp were fed formulated pellet diets (Haid Dachuan #2, Guangdong Haid Group Co., Ltd.), which remain stable as pellets for at least one day, three times per day, at 9:00, 16:00 and 23:00, respectively, and an appropriate feed dose per meal was ensured for their apparent satiation. For each shrimp, feed was stored in an independent container, and unconsumed feed was collected in an independent container at 7:30 every day and then dried until weight was stable. Sea water was exchanged 50% daily and temperature was maintained at 27.5 ± 0.6 °C (mean ± SD). Dead shrimp and molt were removed as soon as possible. The experiment was carried out at the Mariculture Research Station of the Yellow Sea Fisheries Research Institute.

### Data collection

The experiment continued for 42 days and was divided into two successive growth periods, days 1–21 and days 22–42. Basic measures used for the analysis of feed efficiency included body weight (BW) and feed intake (FI). For each shrimp, BW was recorded three times, at the start, middle and end of the experiment (BW1, BW2 and BW3, respectively). BW1 and BW2 were the initial body weight for growth periods days 1–21 and days 22–42, respectively. Weight gain (WG) for each 21-day interval was calculated, i.e., WG1 for days 1–21 and WG2 for days 22–42. For each shrimp, the uneaten feed was collected daily into a container and accumulated until the end of each growth period. Its weight was equal to the amount of feed unconsumed during the period. The amount of feed that was offered during this period was equal to the difference in weight of the feed container at the start and end of the period. FI was calculated as the difference between the amount of feed offered and feed unconsumed (FI1 for days 1–21 and FI2 for days 22–42). Then, the data on WG and FI were checked to exclude animals with unreasonable data. Exclusion criteria included negative, zero and near-zero weight gains and near-zero feed intakes, which indicated that such animals were possibly unhealthy. Sixteen animals were removed accordingly. ADG (ADG1 for days 1–21 and ADG2 for days 22–42) and DFI (DFI1 for days 1–21 and DFI2 for days 22–42) were calculated as WG and FI divided by 21 days, respectively. In addition, gender and age at the end of each period were recorded for each shrimp.

### Calculation of RFI and FER

In this study, RFI and FER were used as measures of feed efficiency. FER was calculated as the ratio between WG and FI (FER1 for days 1–21 and FER2 for days 22–42). To determine RFI (RFI1 for days 1–21 and RFI2 for days 22–42), expected daily feed intake was calculated as a multiple regression with observed daily feed intake as the dependent variable (Model 1):

$$ {\text{DFI}} = b1 \times MW^{b2} + b3 \times {\text{ADG}} + e,\quad ({\text{Model}}\,1) $$

where *MW* is the mid-weight ($MW1 = 1/2(BW1 + BW2)$ and $MW2 = 1/2(BW2 + BW3)$, *MW* ^*b*2^ is the metabolic mid-weight, DFI and ADG are described as above, *e* is the error, and *b*1, *b*2, and *b*3 are partial regression coefficients. The error term is considered to be RFI. The nonlinear regression procedure of nls in R version 3.3.1 [27] was used to analyze the data for each growth period.

### Statistical analyses

The descriptive statistical analysis of RFI, FER, ADG and DFI during the two growth periods was conducted using R version 3.3.1. The distributions of the traits were checked for normality. A pruned pedigree that included the animals tested plus their ancestors for three generations up the pedigree was used to construct a relationship matrix ${\mathbf{A}}$ (see Additional file 1: Table S1). A univariate animal model was used to obtain the variance estimates for RFI, FER, ADG and DFI. The model used is described as:

$$ {\mathbf{Y}}_{{{\mathbf{ijklm}}}} = \mu + Tank_{i} + Sex_{j} + b*Age_{k} + a_{l} + e_{ijklm} ,\quad \left( {{\text{Model}}\; 2} \right) $$

where ${\mathbf{Y}}_{{{\mathbf{ijklm}}}}$ is the vector of the observed trait, *μ* is the overall mean, *Tank*_*i*_ is the fixed effect of the *i*th culture system, *Sex*_*j*_ is the fixed effect of the *j*th gender, *b* is the regression coefficient, *Age*_*k*_ is the covariate of the *k*th individual age at the end of each period, $a_{l}$ is the random additive genetic effect of the *l*-th individual, $a\sim\left( {0, {\mathbf{A}}\sigma_{a}^{2} } \right)$, where ${\mathbf{A}}$ is the additive genetic relationship matrix among all shrimp, and *e*_*ijklmn*_ is the random residual effect of the *l*th individual, $e \sim \left( {0, {\mathbf{I}}\sigma_{e}^{2} } \right)$. The phenotypic variance (*σ*_*p*_^2^) was the sum of all variance components: *σ*_*p*_^2^ = *σ*_*a*_^2^ + *σ*_*e*_^2^. Heritability was estimated as the ratio between animal genetic variance and phenotypic variance: *h* ^2^ = *σ*_*a*_^2^/*σ*_*p*_^2^. In addition, least square means (LSM) for RFI of 34 families during the two periods were estimated based on a similar model in which *a*_*l*_ was replaced by a family effect.

Phenotypic (genetic) correlations between different traits within each period and between the same trait recorded during two periods were estimated using a bivariate animal model. All effects and covariates used in the bivariate animal model were the same as those in Model 2. Note that the common environmental effect was included neither in the univariate nor in the bivariate animal models since it may obstruct estimation of heritabilities or cause convergence problems.

All (co)variance analyses were conducted with the Gibbs sampler included in the Multiple Trait Gibbs Sampling for Animal Models (MTGSAM) program [28]. MTGSAM was used to generate files of samples and posterior estimates of variance components. The initial number of iterations was arbitrarily obtained using a single chain of 100,000 iterations and a burn-in period of 10,000 iterations with a thinning interval of 30. Convergence diagnosis was conducted using the R software with the Bayesian Output Analysis (BOA) package [29]. The mean of the posterior distribution was used as point estimate for (co)variance components, heritabilities, and correlations. The standard deviation of the posterior distribution was used as an equivalent estimate of the frequentist standard error [30]. The Bayesian confidence interval denoted as high posterior density (HPD) interval was obtained through the BOA package of the R software, and 95% HPD intervals that include 95% of the samples were used in this study. The Z-score was used for testing whether the estimates of heritability and correlation were significantly different between traits and between periods, as described by Nguyen et al. [31].

## Results

### Residual feed intake

After removing dead animals and those with unreasonable data, the final dataset used for analysis included 519 animals over two growth periods (see Additional file 2: Table S2, Additional file 3: Table S3). The model for estimating RFI1 was:

$$ {\text{DFI}}1 = 0.067 \times MW1^{0.526} + 0.4 \times {\text{ADG}}1 + e, $$

and the model for ${\text{RFI}}2$ was:

$$ {\text{DFI}}2 = 0.067 \times MW2^{0.454} + 0.394 \times {\text{ADG}}2 + e. $$

Estimates of regression coefficients in the models for RFI1 and RFI2 were close. The determination coefficients (R^2^) of the models for RFI1 and RFI2 were 0.89 and 0.64, respectively. The range of RFI values was wider during the second than the first period (see Table 1).

**Table 1:** Descriptive statistics of average daily gain, daily feed intake, feed efficiency ratio and residual feed intake during the periods days 1–21 and 22–42, respectively

Period		Trait			
		ADG	DFI	FER	RFI
Days 1–21	Max^a^	0.195	0.270	1.084	0.062
	Min^b^	0.030	0.054	0.250	−0.041
	Mean^c^	0.101	0.156	0.653	0.000
	SD^d^	0.032	0.049	0.116	0.016
	CV^e^	0.320	0.313	0.178	–
Days 22–42	Max	0.224	0.288	1.283	0.092
	Min	0.048	0.076	0.193	−0.068
	Mean	0.129	0.194	0.675	0.000
	SD	0.035	0.040	0.162	0.024
	CV	0.272	0.204	0.239	–

*ADG* average daily gain (g/day), *DFI* daily feed intake (g/day), *FER* feed efficiency ratio (g/g), *RFI* residual feed intake (g/day)

^a^Maximum of the trait estimates

^b^Minimum of the trait estimates

^c^Mean of the trait estimates

^d^Standard deviations for the trait estimates

^e^Coefficient of variation for the trait estimates (%)

**Fig. 1:**
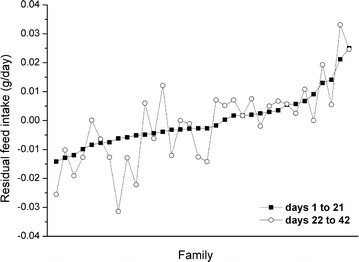
Least square means of 34 families for residual feed intake (RFI) during days 1–21 and days 22–42 of the test period

LSM for RFI of 34 families during the two periods are in Fig. 1. The RFI of the families ranged from –0.014 to 0.025 g/day for the first period and displayed substantial inter-family variation. Although the LSM of RFI differed between the two periods for most families, the ranking of the 34 families based on RFI tended to be similar across the two periods (Spearman’s correlation coefficient = 0.73, *P* < 0.01).

### Descriptive statistics of other traits

Descriptive statistics of ADG, DFI and FER are also in Table 1. The coefficients of variation (CV) show that shrimp displayed large inter-individual variation in these traits during each growth period. The average values of FER, ADG and DFI increased significantly between the first and second period (*P* < 0.01) but the CV of FER increased notably while those of ADG and DFI declined dramatically.

### Variance components and heritability

Posterior mean estimates and 95% HPD intervals of variance components and heritabilities for the four traits during the two growth periods are in Table 2. Estimates of heritabilities of RFI were high (0.641 ± 0.237 for period 1 and 0.707 ± 0.252 for period 2), with no significant differences between periods (*P* > 0.05). Estimates of heritabilities of FER, as another measure of feed efficiency, were also high (0.577 ± 0.232 and 0.688 ± 0.253 for the two periods). There were no significant differences in heritability between RFI and FER (*P* > 0.05) within each period. In addition, ADG and DFI were highly heritable (0.598 ± 0.255 to 0.696 ± 0.239) across the two periods.

**Table 2:** Posterior means and 95% high posterior density intervals of variance components and heritabilities for average daily gain, daily feed intake, feed efficiency ratio and residual feed intake during the two growth periods

Period	Trait	Variance component			Heritability	
		*σ*_*a*_^2^	*σ*_*e*_^2^	*σ*_*p*_^2^	Mean ± SD	HPD
Days 1–21	ADG	6.52E−04	3.69E−04	1.02E−03	0.638 ± 0.242	0.390–0.903
	DFI	1.24E− 3	5.43E−04	1.78E−03	0.696 ± 0.239	0.410–0.899
	FER	7.62E−03	5.59E−03	1.32E−02	0.577 ± 0.232	0.345–0.882
	RFI	1.55E−04	8.72E−05	2.43E−04	0.641 ± 0.237	0.392–0.905
Days 22–42	ADG	7.34E−04	4.94E−04	1.23E−03	0.598 ± 0.255	0.363–0.928
	DFI	9.16E−04	4.63E−04	1.38E−03	0.664 ± 0.247	0.361–0.960
	FER	1.65E−02	7.49E−03	2.40E−02	0.688 ± 0.253	0.429–0.937
	RFI	3.38E−04	1.41E−04	4.79E−04	0.707 ± 0.252	0.460–0.945

*ADG* average daily gain (g/day), *DFI* daily feed intake (g/day), *FER* feed efficiency ratio (g/g), *RFI* residual feed intake (g/day), *SD* standard deviation, *HPD* high posterior density interval at the 95% level

### Trait correlations

Posterior means and 95% HPD intervals of the phenotypic and genetic correlations between traits within each period are in Table 3. FER had high positive genetic correlations with ADG for both periods (0.624 ± 0.280 and 0.851 ± 0.100). In contrast, RFI showed a weak genetic correlation with ADG during days 1–21 (0.135 ± 0.204) and no genetic correlation with ADG during days 22–42 (–0.018 ± 0.128). These two feed efficiency traits had weak and moderate negative genetic correlations during days 1–21 and days 22–42, respectively (–0.126 ± 0.208 and –0.387 ± 0.183). Both RFI and ADG had high positive genetic correlations with DFI across the two periods. Overall, genetic correlations between any two traits were not significantly different between the two periods (*P* > 0.05).

**Table 3:** Posterior means and 95% high posterior density intervals, of the phenotypic and genetic correlations between traits within each period

Correlation	Period	Trait					
		${\text{ADG}} - {\text{FER}}$ ^a^	${\text{ADG}} - {\text{RFI}}$ ^b^	${\text{FER}} - {\text{RFI}}$ ^c^	${\text{DFI}} - {\text{RFI}}$ ^d^	${\text{DFI}} - {\text{FER}}$ ^e^	${\text{ADG}} - {\text{DFI}}$ ^f^
Phenotypic	Days 1–21	0.404 ± 0.037 (0.329, 0.467)	–0.007 ± 0.048 (–0.055, 0.052)	–0.613 ± 0.028 (–0.650, –0.579)	0.403 ± 0.042 (0.324, 0.475)	–0.238 ± 0.042 (–0.316, –0.152)	0.815 ± 0.015 (0.790, 0.843)
	Days 22–42	0.682 ± 0.024 (0.640, 0.720)	0.005 ± 0.040 (–0.042, 0.091)	–0.514 ± 0.030 (–0.567, –0.469)	0.628 ± 0.028 (0.583, 0.669)	–0.203 ± 0.051 (–0.299, –0.108)	0.543 ± 0.032 (0.482, 0.588)
Genetic	Days 1–21	0.624 ± 0.280 (0.333, 0.998)	0.135 ± 0.204 (–0.130, 0.377)	–0.126 ± 0.208 (–0.413, 0.115)	0.815 ± 0.169 (0.590, 1)	–0.709 ± 0.291 (–1, –0.499)	0.732 ± 0.272 (0.510, 0.989)
	Days 22–42	0.851 ± 0.100 (0.714, 0.999)	–0.018 ± 0.128 (–0.204, 0.225)	–0.387 ± 0.183 (–0.575, –0.209)	0.868 ± 0.098 (0.740, 0.999)	–0.765 ± 0.300 (–0.995, –0.452)	0.586 ± 0.138 (0.435, 0.746)

The lower and upper limits of the 95% high posterior density intervals are indicated in brackets

^a^Correlation between average daily gain and feed efficiency ratio

^b^Correlation between average daily gain and residual feed intake

^c^Correlation between feed efficiency ratio and residual feed intake

^d^Correlation between daily feed intake and residual feed intake

^e^Correlation between daily feed intake and feed efficiency ratio

^f^Correlation between average daily gain and daily feed intake

### Correlations between periods

Posterior means and 95% HPD intervals of the phenotypic and genetic correlations for the same traits recorded in the two periods are in Table 4. For each trait, the genetic correlation between the two periods was high and positive (>0.6), which suggests that traits tended to be consistent across periods in spite of some variation. The magnitude of correlations between periods did not differ significantly between traits (*P* > 0.05).

**Table 4:** Posterior means and 95% high posterior density intervals of the phenotypic and genetic correlations between the same traits recorded during days 1–21 and 22–42

Correlation	${\text{ADG}}1 - {\text{ADG}}2$ ^a^	${\text{DFI}}1 - {\text{DFI}}2$ ^b^	${\text{FER}}1 - {\text{FER}}2$ ^c^	${\text{RFI}}1 - {\text{RFI}}2$ ^d^
Phenotypic	0.407 ± 0.134 (0.207, 0.614)	0.706 ± 0.022 (0.664, 0.744)	0.264 ± 0.044 (0.189, 0.353)	0.386 ± 0.037 (0.316, 0.463)
Genetic	0.666 ± 0.163 (0.470, 0.838)	0.781 ± 0.130 (0.623, 0.940)	0.799 ± 0.137 (0.617, 0.962)	0.714 ± 0.219 (0.445, 0.978)

The lower and upper limits of the 95% high posterior density intervals are indicated in brackets

^a^Correlation for average daily gain between periods

^b^Correlation for daily feed intake between periods

^c^Correlation for feed efficiency ratio between periods

^d^Correlation for residual feed intake between periods

## Discussion

Although feed efficiency is an economically important trait for shrimp production, to date it has not been studied for shrimp selection programs. The challenges associated with measuring individual feed intake have prevented accurate estimation of individual genetic values for feed efficiency. The X-ray method, which has been widely used in fish, is probably unsuitable for shrimp, since their eating habits and digestion differ from those of fish. Thus, in this study we measured individual feed intake directly by housing shrimp in isolation. Good management is the first requirement for individual housing on a large scale, because rearing many animals in a re-circulating system is difficult to control and feeding them successively is labor-intensive and time-consuming. Although currently individual housing appears to be the most feasible way of measuring individual feed intake in shrimp, implementing such a test on a large scale is still a demanding process.

RFI is a trait that is now widely used to study the genetic variability of feed efficiency and the physiological mechanisms that underlie this variation, mainly in endothermic land vertebrates. According to Luiting and Urff [32], two models for relating feed intake to expected maintenance requirements are available to calculate RFI: (1) an approximation by inclusion of an intercept into the model, along with a regular metabolic body weight exponent, and (2) a nonlinear approximation in which the exponent is estimated. A model with an intercept of zero and a regular metabolic body weight exponent of 0.75 (MW^0.75^) is generally used for terrestrial agricultural animals including cattle, pigs and poultry. However, exponents deviating from 0.75 were also reported for these species, e.g. 0.5, 1.0 and 1.2 in laying hens [33]. In fish, the most frequently used exponent for metabolic body weight is 0.8 [34]. Since many physiological mechanisms differ between shrimp and livestock species, it is not possible to extrapolate the exponent of metabolic body weight used for those species to the model for shrimp. Thus, in this study, we used the nonlinear approximation to estimate the metabolic body weight exponents in the model for RFI. Compared with those for other species, the lower estimates of the exponents found here (0.526 and 0.454 for periods 1 and 2) may be related to the poikilothermism and smaller size of *L. vannamei*.

Having an estimated heritability for RFI during the grow-out period is critical for future genetic selection on this trait in the breeding population of *L. vannamei*. This is the first study that evaluates genetic parameters of RFI in shrimp, and the estimated heritabilities of RFI during the two successive growth periods (0.641 ± 0.237 and 0.707 ± 0.252, respectively) were higher than most of the previously published estimates in other species, ranging from 0.18 to 0.64 in cattle [12, 35–37], from 0.18 to 0.47 in pigs [15, 38, 39], from 0.21 to 0.83 in poultry [40–42], and 0.29 in fish [9]. These heritabilities are most likely overestimated, in part because the common environmental effects could not be partitioned out. When family (the common environmental effect) was included as a random effect in the univariate animal models, the variance components of animal or family tended to be equal to zero in most cases. The weak genetic relationships between families due to the lack of half-sib families (there were few half-sib families among the 34 full-sib families) and the shallow depth of the pedigree did not allow the common environmental effects to be partitioned out [43, 44]. Convergence problems existed when family was included in the bivariate animal models, which means that the relatively small number of families and animals tested in this study was not sufficiently informative for models with many variance parameters. In addition, the small sample size resulted in large standard deviations and 95% probability intervals for the estimated heritabilities.

In fish, social interactions such as dominance hierarchies are recognized as an important cause of variation in feed intake, with dominant individuals exhibiting superior growth and appetite relative to subordinates [45]. This is also the case in shrimp. It seems inevitable that possible genotype by environment interactions between individual-housed and group-housed situations would lead to some biases in the heritability estimates. The lack of social interactions in the individual-housed situation may reduce the phenotypic variation in feed intake among individuals and substantially the environmental (error) variation, which to some degree could cause the overestimation of heritabilities for related traits. From another perspective, individual differences in growth and feed behavior in absence of social interactions are most likely to be inherent and genetically linked [21]. Nevertheless, the heritabilities estimated here still indicate significant genetic variability of RFI in *L. vannamei*.

Trait estimates were different between the two successive growth periods. Shrimp need to adapt to the individual-housed situation during the initial period, with a modulation of physiological responses, and this may contribute to the difference in trait estimates between the two periods. Moulting is also an important factor that affects the feeding behavior of shrimp. Compared with terrestrial animals and fish, in shrimp larger day-to-day variation in feed intake exists over the entire production period. However, genetic correlations between the two periods for each trait RFI, FER, ADG and DFI still reached high levels (>0.6), which indicates that, in shrimp, trait estimates tended to be consistent across at least three-week periods. Moreover, trait heritabilities and genetic correlations did not differ significantly between the two periods (*P* > 0.05). These results indicate that a three-week testing period is probably sufficient for accurate measurement of feed efficiency traits.

In this study, the genetic correlation of FER was high with both ADG and DFI, which is expected due to autocorrelation effects. High correlations between feed efficiency (FER or its reciprocal) and growth rate were also reported in previous studies on terrestrial animals and fish [42, 46–48], which suggests that FER can be indirectly improved by selection for fast growth. However, according to the findings of Quinton et al. [23], the genetic response in FER by selection on growth alone in fish was at least two times slower than that by simultaneous selection for fast growth and reduced feed intake. However, in *L. vannamei,* the high positive genetic correlation between feed intake and growth rate may not be favorable for simultaneous selection for fast growth and reduced feed intake.

In contrast, weak or no genetic correlations between RFI and ADG across the two periods indicated that selecting for feed efficiency using RFI is not expected to influence growth performance in *L. vannamei*. This result is in agreement with the findings on other species [14, 41, 49]. The high positive genetic correlations between RFI and DFI are likely favorable, because selecting for low RFI will decrease feed intake. Although multiple-trait selection on RFI and production has been reported to be equivalent to that on feed intake and production in cattle [13], unlike RFI, feed intake is difficult to interpret as a stand-alone trait independent of growth rate. Weak and moderate genetic correlations were observed between RFI and FER across the two periods (–0.126 ± 0.208 and –0.387 ± 0.183), which were lower than those reported in other species (–0.23 to –0.66) [11, 49–52]. These estimates also indicate that a selection scheme that uses RFI or FER as criteria may have a different selection response.

## Conclusions

Accurate measurement of individual feed efficiency on a large scale was achieved for the first time in shrimp. We show that RFI is a heritable trait in *L. vannamei*, which confirms that genetic improvement of this trait is possible. However, this result must be considered with caution since the heritability estimates may be overestimated. For *L. vannamei*, the biggest potential advantage of using RFI as a measure of feed efficiency is that it is independent of growth rate, and thus genetic selection against RFI has the potential to improve feed efficiency and reduce feed intake, without compromising growth performance. Genetic improvement for feed efficiency can be achieved by including RFI in a multiple-trait selection index. In beef cattle, the estimated economic benefits are considered to improve profit by 9 to 33% when including RFI in the multiple-trait selection [53]. Further analysis of the selection index is necessary to determine the relative weight for RFI in the current index for *L. vannamei* before it can be successfully incorporated into selection programs.

## Additional files

**Additional file: Table S1.** A pruned pedigree including animal ID, sire ID and dam ID.

**Additional file: Table S2.** Information and phenotypic data for animals tested during the period from day 1 to 21. Data include animal ID, sire ID, dam ID, family ID, sex, tank ID and the age, and phenotypic data include initial body weight (IBW), feed efficiency ratio (FER), average daily gain (ADG), daily feed intake (DFI) and residual feed intake (RFI).

**Additional file: Table S3.** Information and phenotypic data for animals tested during the period from day 22 to 42. Data include animal ID, sire ID, dam ID, family ID, sex, tank ID and the age, and phenotypic data includes initial body weight (IBW), feed efficiency ratio (FER), average daily gain (ADG), daily feed intake (DFI) and residual feed intake (RFI).
